# Assessment of treatment expectations in people with suspected endometriosis: A psychometric analysis

**DOI:** 10.12688/f1000research.145377.2

**Published:** 2024-09-09

**Authors:** Ann-Katrin Meyrose, Lukas A. Basedow, Nina Hirsing, Olaf Buchweitz, Winfried Rief, Yvonne Nestoriuc

**Affiliations:** 1 Clinical Psychology and Psychotherapy, Helmut-Schmidt-University / University of the Federal Armed Forces Hamburg, Hamburg, Germany; 2 Department of Child and Adolescent Psychiatry, Psychotherapy, and Psychosomatics, University-Medical Center Hamburg-Eppendorf, Hamburg, Germany; 3 Division of Clinical Psychology and Psychotherapy, Department of Psychology, Philipps-Universitat Marburg, Marburg, Germany; 4 Frauenklinik an der Elbe, Center of Surgical Endoscopy and Endometriosis, Hamburg, Germany; 5 Institute of Systems Neuroscience, University-Medical Center Hamburg-Eppendorf, Hamburg, Germany

**Keywords:** placebo, nocebo, expectancies, laparoscopy, G-EEE, TEX-Q

## Abstract

**Background:**

Treatment expectations influence clinical outcomes in various physical and psychological conditions; however, no studies have explored their role in endometriosis treatment. It is necessary to understand how these expectations can be measured to study treatment expectations and their effects in clinical practice. This study aimed to psychometrically analyze and compare different treatment expectation measurements and describe treatment expectations in women with suspected endometriosis.

**Method:**

Analysis of cross-sectional baseline data of a mixed-method clinical observational study of *N*=699 patients undergoing laparoscopy in Germany. Descriptives, bivariate associations, convergent and discriminant validity of four expectation measurements (Treatment Expectation Questionnaire (TEX-Q); Generic rating scale for previous treatment experiences, treatment expectations, and treatment effects (GEEE); numerical rating scales (NRS) assessing improvement and worsening of endometriosis symptoms, expected Pain Disability Index (PDI); range: 0 to 10) were estimated. A cluster analysis was performed on the three GEEE items.

**Results:**

Most participants expected high improvement ( *M*=6.68 to 7.20, *SD*=1.90 to 2.09) and low worsening ( *M*=1.09 to 2.52, *SD*=1.80 to 2.25) of disability from laparoscopy. Participants who expected greater worsening expected more side effects ( *r*=.31 to .60, *p*<.001). Associations between the positive and negative expectation dimensions, including side effects, were small to non-significant ( *r* =|.24| to .00, *p*<.001 to.978). Four distinct clusters, described as’positive’, ‘no pain, no gain’, ‘diminished’, and ‘uniform’ were found, with a total PVE of 62.2%.

**Conclusions:**

Women with suspected endometriosis reported positive expectations concerning laparoscopy, but wide ranges indicated interindividual differences. Treatment expectations seem to be a multidimensional construct in this patient group. The investigated measurements did not correlate to the extent that they measured exactly the same construct. The selection of measurements should be carefully considered and adapted for the study purposes. Clusters provide initial indications for individualized interventions that target expectation manipulation.

**Trial Registration Number:**

ID NCT05019612 ( ClinicalTrials.gov)

## Introduction

Treatment expectations are important for clinical outcomes in various physical and psychological conditions, ^1^ such as acute and chronic pain, ^2^^,^^3^ surgeries, ^4^^,^^5^ breast cancer, ^6^ and psychiatric disorders. ^7^ For pain and depression effects were large: Up to 70% of treatments effects can be attributed to expectations. For analgesic treatments, up to 50% of treatment effect can be attributed to placebo effects. ^8^^,^^9^ Auer et al. (2016) found low to moderate associations between presurgery patients’ expectations and postsurgery quality of life. Despite the high prevalence of endometriosis (4.4% in the general population ^10^) and enormous individual burden, ^11^ no study has focused on treatment expectations in people with suspected endometriosis. Endometriosis is a chronic inflammatory disease in women of procreative age and is characterized by endometrium-like tissues outside the uterus. ^12^ The five most prevalent symptoms of endometriosis are dysmenorrhea, abdominal pain, dyspareunia, dyschezia, and dysuria. ^13^ The German S2k-treatment guideline for endometriosis recommends a laparoscopy, a minimally invasive surgery under general anesthesia, when endocrine therapy has failed. ^14^ Laparoscopy is associated with short-term symptom improvement, ^15^ but 20–30% of patients do not respond satisfactorily. ^16^^,^^17^ Depressive symptoms, anxiety, and pain catastrophization seem to be associated with persistent symptoms. ^18^^–^^20^ In summary, current treatment options are inadequate for many patients. ^21^ A deeper understanding of the role of treatment expectations might help a) clarify why complaints persist and b) develop new intervention avenues to foster positive expectations and prevent nocebo effects. ^22^

### Measurement of expectations

To properly implement and study treatment expectations and their effects in clinical practice, it is necessary to have a good understanding of how these expectations can be measured. This is not as easy as it seems, since treatment expectations can and should be considered multidimensional constructs that differ across specific contexts and time intervals. ^23^ Specifically, expectations can be positive (e.g., improvement of symptoms) or negative (e.g., worsening of symptoms or side effects), ^24^ related to treatment processes or outcomes, ^25^ rated based on valence (more or less important) or probability of occurrence, ^26^ treatment-specific or related to general symptoms, ^27^ or different in terms of being real, that is, plausible or ideal expectations. ^23^^,^^25^ This complexity of the ‘treatment expectation’ construct leads to pronounced heterogeneity in available instruments. ^28^^–^^30^ While single-item measures have been the most popular historically, ^26^^,^^29^^,^^31^ recent advances have led to the development of multidimensional treatment-expectation questionnaires. ^28^^,^^32^ Although this development circumvents several issues in the assessment of treatment expectations, ^23^ an ideal method of expectation measurement has not been developed. Several issues remain understudied and are fruitful targets for continued psychometric research, such as the contrast between context specific and general expectations.

### Aims and hypotheses

The first aim of this study was to describe and exploratively cluster pre-operative treatment expectations in this burdened and understudied patient group. The second aim was to psychometrically analyze and compare different treatment expectation measurements in this large sample of N=699 people with suspected endometriosis. Referring to our second aim, we hypothesise that:

i) Treatment expectations are a multidimensional construct; that is, intercorrelations of different scales and dimensions of the *Treatment Expectation Questionnaire* (TEX-Q) ^28^ and the three expectation items of the *Generic rating scale for previous treatment experiences, treatment expectations, and treatment effects* (GEEE ^32^) will be heterogeneous.
ii) Treatment expectation measurements correlate higher with each other in terms of convergent validity compared to other psychological measurements assessed (i.e., disability, severity of symptoms, depressive symptoms, anxiety, and catastrophizing pain) in terms of discriminant validity.

## Methods

This study used pre-operative data from the baseline assessment of a mixed-method clinical observational study ^33^ that focused on patients undergoing laparoscopy for suspected endometriosis. People with endometriosis reported their endometriosis-related disability, complaints, and a priori specified predictors once before and eight times after laparoscopy, covering trajectories over a 12-month period. This observational study was registered at ClinicalTrials.gov (ID NCT05019612), but baseline analyses were not described.

### Participants

The target population included people with endometriosis-related complaints with or without an unmet wish to have children aged 18 and older. Further inclusion criteria were sufficient German language skills, female sex, informed consent for study participation, and indications for laparoscopy. For these analyses, *N*=699 patients were included, irrespective of the actual surgery or clinical diagnosis after laparoscopy.

### Recruitment and procedure

People with an appointment for laparoscopy in a specialized center for surgical endoscopy and endometriosis (Frauenklinik an der Elbe, Germany) were informed about the study by phone. If interested, the people were referred to the baseline online survey. The online survey included written information about the study and checked the inclusion criteria. Written informed consent for the online survey, storage and processing of data was obtained online from all participants, and baseline assessment was initiated. An individualized study code was used to store the data. Patients completed the survey between 25 ^th^ of August 2021 to 27 ^th^ of June 2023 until the required sample size of longitudinally participating patients with diagnosed endometriosis was reached. Interested patients were sent email reminders to encourage their participation. Detailed information on the study design, postoperative assessments, and further efforts to address potential bias are described in the study protocol. ^33^

The authors assert that all procedures contributing to this work comply with the ethical standards of the relevant national and institutional committees on human experimentation and the Helsinki Declaration of 1975, as revised in 2008. All procedures involving patients were approved by the Psychotherapeutenkammer Hamburg, Germany (ROXWELL-2021-HH, 25 ^th^ of June 2021).

### Measurements

Treatment expectations about laparoscopy were assessed by four self-reported measurements (Treatment Expectation Questionnaire; three items assessing treatment expectations of the Generic rating scale for previous treatment experiences, treatment expectations, and treatment effects; self-constructed numerical rating scales; and expected Pain Disability Index). Table 1 gives an overview of expectation measurements. The Treatment Expectation Questionnaire (TEX-Q) ^28^^,^^34^ comprises 15 items of six dimensions with 11 response options each. The mean score ranged from 0 to 10. Higher scores indicated more positive treatment expectations for the total score and dimensions of ‘treatment benefit’, ‘positive impact’, ‘process’, and ‘behavioral control’. Higher scores indicate more negative treatment expectations for the ‘adverse events’ and ‘negative impact” dimensions. In this study, Cronbach’s α of the total score was.82 and ranged between.72 and.91 for the dimensions in accordance with the validation sample. ^34^

**Table 1.** Overview of expectation measurements under analysis.

Expectation measurement	Treatment expectation questionnaire	Generic rating scale for treatment expectations ^**a**^	Numerical rating scales	Expected pain disability index
Abbreviation	TEX-Q	GEEE	NRS	Expected PDI
Reference	^28^^,^^34^	^32^	Self **-**constructed	Adapted PDI by study team ^35^^,^^36^
Number of items	15	3	10	7
Response options	11 (range: 0-10)	11 (range: 0-10)	11 (range: 0-10)	11 (range: 0-10)
Dimensions	1. treatment benefit 2. positive impact 3. adverse events 4. negative impact 5. process 6. behavioral control + Total score	expected improvement of disability expected worsening of disability expected side effects from laparoscopy	expected improvement and worsening of dysmenorrhea, pelvic/abdominal pain, dyspareunia, dyschezia, dysuria	expected disabilities after laparoscopy

^a^ Three items assessed to measure treatment expectations of the Generic rating scale for previous treatment experiences, treatment expectations, and treatment effects.

Three items assessing treatment expectations of the Generic rating scale for previous treatment experiences, treatment expectations, and treatment effects (GEEE) ^32^ were used, focusing on expected improvement of disability, worsening of disability, and side effects from laparoscopy with 11 response options (0=no improvement/impairment to 10=greatest improvement/worsening imaginable; 0=no complaints to 10=greatest complaints imaginable). No sum or mean scores were obtained.

Ten self-constructed numerical rating scales (NRS) with 11 response options were used to assess the expected improvement and worsening of the five most prevalent endometriosis symptoms (dysmenorrhea, pelvic/abdominal pain, dyspareunia, dyschezia, and dysuria). The wording of items and response options (0=no improvement/impairment to 10=greatest improvement/worsening imaginable) were derived from the GEEE in accordance with the recommendations for the assessment of pain. No sum or mean scores were obtained.

Expected endometriosis-related pain disability was assessed using the German version of the Pain Disability Index ^35^^,^^36^ adapted to expected disabilities after laparoscopy (expected PDI). The expected PDI covers seven items with 11 response options (0=no disability to 10=total disability). The sum (range: 0-70) and mean scores (0-10) were calculated. Higher scores indicate higher expected disability. The mean scores were mainly used because of their better comparability to the other expectation measurements in this study. The Cronbach’s α was.93 in this study. The expected change in disability by laparoscopy was defined as the difference between endometriosis-related disability before laparoscopy and expected endometriosis-related disability after laparoscopy (ΔPDI score=PDI – expected PDI; theoretically ranging from -70 to 70 for the sum and -10 to 10 for the mean score). Positive scores indicate a positive change in disability by laparoscopy, that is, improvement, whereas negative scores indicate a negative change in disability, that is, worsening.

Further, self-reported psychological measurements were used to describe the sample and estimate discriminant validity. Endometriosis-related disability was assessed using the PDI ^35^^,^^36^ covering seven items with 11 response options (0=no to 10=total disability). The introductory text was adapted to *endometriosis-related* disability. The sum (range: 0-70) and mean scores (0-10) were calculated. Higher scores indicate higher disability. Cronbach’s α was .85 in this study. Disability will be compared to women of the German general population. ^37^

The severity of endometriosis-related symptoms was assessed by five NRS referring to the five most prevalent endometriosis symptoms (dysmenorrhea, pelvic/abdominal pain, dyspareunia, dyschezia, and dysuria) with 11 response options (0=no pain to 10=worst pain imaginable). To summarize symptoms, the maximum severity of symptoms score was calculated using the stated maximum severity of symptoms within the five NRS, for example, if someone selected response options between 2 and 8 for the five endometriosis symptoms, the maximum severity of symptoms score was 8.

Depressive symptoms and anxiety were assessed using the Patient Health Questionnaire (PHQ-4) ^38^ which covers four items with four response options (0=not at all, 1=several days, 2=more than half the days, 3=nearly every day). Sum scores for the depressive symptoms and anxiety subscales ranged from 0 to 6. Higher scores indicate more depressive symptoms and anxiety, respectively. Cronbach’s α was .77 for depressive symptoms and.76 for anxiety in this study. A cutoff score of 3 or greater indicates good sensitivity and specificity for detecting major depression ^39^ and anxiety disorders. ^40^

Pain catastrophizing was assessed by the subscale catastrophizing of the Coping Strategies Questionnaire (CSQ) ^41^ which covers six items with seven response options each (0=never do that to 6=always do that). The sum score ranges from 0 to 36, with higher scores indicating a higher level of catastrophizing pain. The Cronbach’s α was .88 in this study.

Further, following self-reported medical characteristics were used to describe the sample: reason for laparoscopy, duration of endometriosis symptoms (in months), previous experience with laparoscopy (yes/no), current endocrine therapy (yes/no), and whether complaints depend on menstrual bleeding (before, during, after, independent).

### Statistical analysis

Frequencies and descriptive statistics (mean, standard deviation, median, range, skewness, and kurtosis) were calculated to describe the sample. Missing data are also reported. Distributions of treatment expectation measurements are displayed by raincloud plots using the R packages *ggplot2*, *ggdist, ggforce, gghalves, haven, RcolorBrewer.* Bivariate associations were estimated using Pearson correlation coefficients and displayed as a correlation matrix heatmap using the R package ggcorrplot. ^42^ Convergent validity was determined by bivariate associations of all treatment expectation measurements, discriminant validity by bivariate associations of treatment expectation measurements, and psychological constructs. According to Cohen, ^43^ Pearson’s *r*=.1–.3 is interpreted as *small*, *r*=.3–.5 as *medium*, and r≥.5 as *large.* The two-sided level of significance was set at α=.05. The optimal number of clusters was determined by visual inspection of elbow and silhouette plots. The resulting optimal number of clusters was used for the k-means clustering of the three G-EEE items using the *cluster* package. ^44^ SPSS version 27 and R version 2022.12.0+353 were used for statistical analyses. The data that support the findings of this study are openly available. ^45^

## Results

### Descriptive characteristics

**Figure 1.**
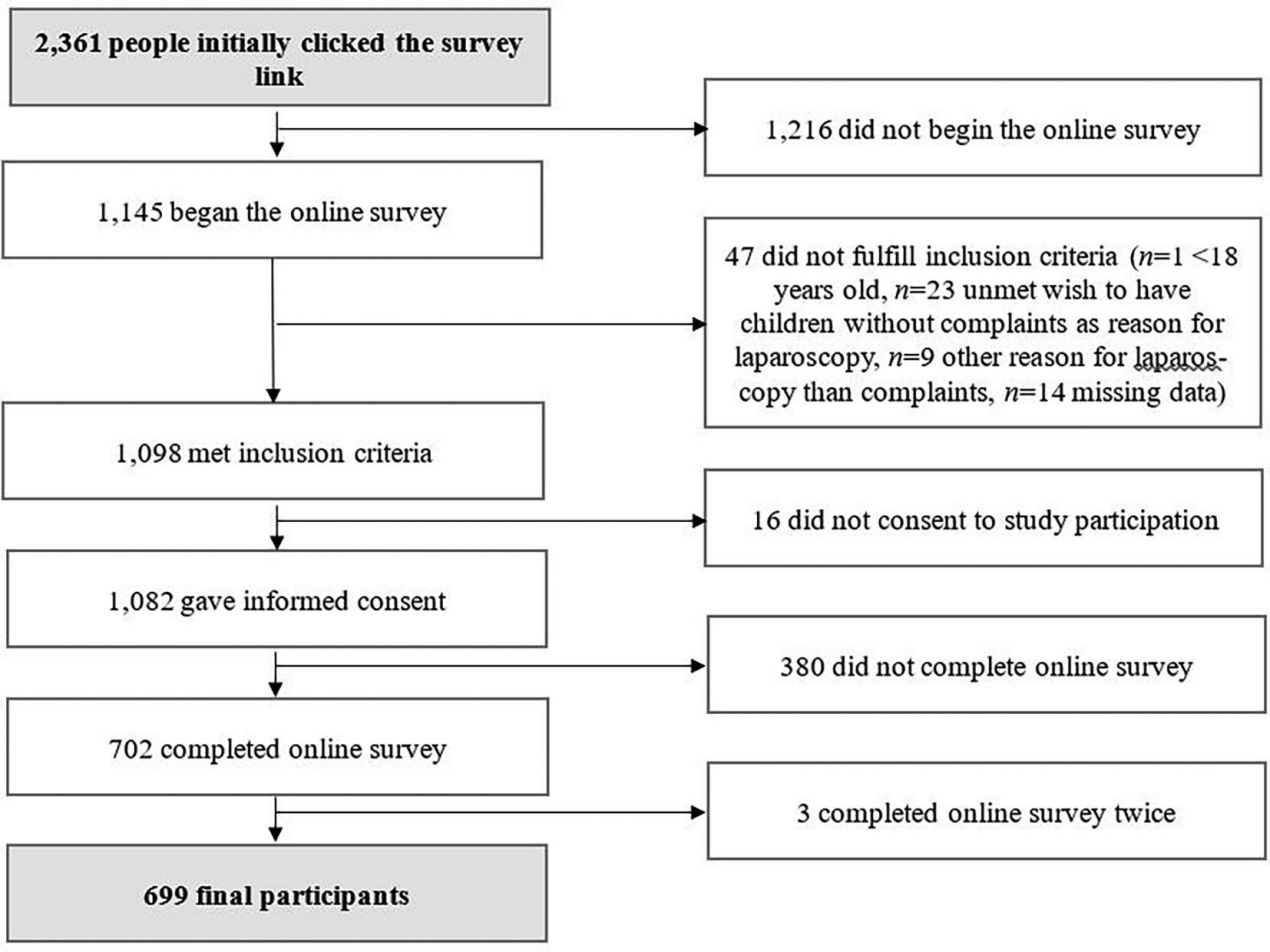
Flow chart of study participation.

Overall, *n*=2,361 people clicked the survey link, *n*=1,145 began the online survey, *n*=1,098 met the inclusion criteria, *n*=1,082 gave informed consent, and *n*=699 completed the online survey. More details on the flow of participants are displayed in Figure 1.

The ages of the 699 participants with suspected endometriosis ranged from 18 to 51 years ( *M*=29.90, *SD*=6.45). About one-fifth had a migrant background, i.e. participants or one or both parents were not born in Germany, and 39.2% had acquired a higher entrance certificate (German ‘Abitur’) and 33.2% had a university degree. The reason for laparoscopy was endometriosis-related complaints with (21.5%) or without (77.5%) an unmet wish to have children. Overall, participants were enormously burdened by endometriosis-related symptoms before laparoscopy (PDI sum score: *M*=30.52, *SD*=13.66) compared with women in the general population in Germany ( *M*=6.9, *SD*=11.1, *N*=1,368 ^37^; *d*=3.14). On average, the maximum severity within the five endometriosis-related symptom scales (NRS) was *M*=7.18 ( *SD*=1.81, potential range: 0-10). More than half (54.8%) showed signs of major depression (41.6%, *M*=2.56, *SD*=1.40) and/or anxiety disorder (42.6%, *M*=2.44, *SD*=1.63) according to the PHQ-4. Approximately one-fifth had previous experience with laparoscopy (21.3%) and one-fifth was currently treated with endocrine therapy (20.3%). Table 2 presents more information on the characteristics of the sample under analysis and the descriptive statistics of all the treatment expectation measurements.

**Table 2.** Descriptive characteristics of the sample under analysis ( *N*=699).

	*n (%)*	*M*	*SD*	*Md*	Observed range	Potential range	Skew	Kurt
**Gender**								
Female	686 (98.1)							
Non-binary	13 (1.9)							
**Age** in years	699	29.90	6.45	29.50	18-51	≥18	0.47	-0.21
**Migrant background**								
Yes	129 (22.5)							
No	445 (77.5)							
**Educational level**								
No school degree	2 (0.4)							
In school	8 (1.1)							
Lower secondary school	13 (1.9)							
Secondary school	170 (24.3)							
Higher entrance certificate	274 (39.2)							
University degree	232 (33.2)							
**Able to work despite endometriosis-related complaints**								
Yes	623 (89.1)							
No	76 (10.9)							
**Reason for laparoscopy**								
Complaints	549 (77.5)							
Complaints with an unmet wish to have children	150 (21.5)							
**Duration of symptoms** in months	684 (97.9)	77.48	78.39	48.00	2-468	>0	1.42	1.61
**Previous experience with laparoscopy**								
Yes	149 (21.3)							
No	550 (78.7)							
**Current endocrine therapy**								
Yes	142 (20.3)							
No	557 (79.7)							
**Dependency of complaints on menstrual bleeding**								
Before	107 (15.3)							
During	298 (42.6)							
After	20 (2.9)							
Independent	274 (39.2)							
**Severity of symptoms (NRS)**								
Dysmenorrhoea ^a^	624 (89.3)	6.85	1.95	7.00	0-10	0-10	-0.80	0.51
Abdomen	699	5.72	2.27	6.00	0-10	0-10	-0.50	-0.32
Sexual intercourse ^b^	476 (68.1)	3.61	2.72	3.50	0-10	0-10	0.31	-0.85
Dysuria	699	1.43	2.10	0.00	0-10	0-10	1.50	1.48
Dyschezia	699	2.52	2.60	2.00	0-10	0-10	0.76	-0.44
Max. severity of symptoms	699	7.18	1.81	8.00	1-10	0-10	-0.82	0.55
**Disability (PDI)**								
Total sum score	699	30.52	13.66	31.00	0-70	0-70	0.01	-0.31
Total mean score	699	4.36	1.95	4.43	0-10	0-10	0.01	-0.31
**Depressive symptoms (PHQ-4)**	699	2.56	1.40	2.00	0-6	0-6	0.53	-0.03
**Anxiety (PHQ-4)**	699	2.44	1.63	2.00	0-6	0-6	0.43	-0.53
**Catastrophizing pain (CSQ)**	699	18.26	6.91	18.00	6-35	0.36	0.25	-0.62
**TREATMENT EXPECTATIONS**								
**TEX-Q**								
Total score	699	6.73	1.28	6.73	2.40-10	0-10	-0.19	0.03
Treatment benefit	699	7.15	1.90	7.33	0-10	0-10	-0.67	0.20
Positive impact	699	6.68	2.35	7.00	0-10	0-10	-0.64	-0.17
Adverse events	699	3.67	1.93	3.33	0-9.67	0-10	0.34	-0.34
Negative impact	699	2.52	2.25	2.00	0-10	0-10	0.80	-0.08
Process	699	6.89	1.92	7.00	0-10	0-10	-0.30	-0.32
Behavioral control	699	5.87	2.44	6.00	0-10	0-10	-0.33	-0.52
**Generic rating scales (GEEE)**								
Improvement	699	7.20	2.09	8.00	0-10	0-10	-0.84	0.67
Worsening	699	1.09	1.79	0.00	0-10	0-10	2.17	5.21
Side effects	699	3.72	2.33	3.00	0-10	0-10	0.30	-0.62
**Expected severity of symptoms (NRS)**								
Dysmenorrhoea improvement ^a^	624 (89.3)	6.70	2.34	7.00	0-10	0-10	-0.73	0.19
Dysmenorrhoea worsening ^a^	624 (89.3)	0.0.92	1.79	0.00	0-10	0-10	2.68	8.10
Abdomen improvement	699	6.94	2.35	7.00	0-10	0-10	-0.86	0.51
Abdomen worsening	699	1.05	2.01	0.00	0-10	0-10	2.50	6.21
Sexual intercourse improvement ^b^	476 (68.1)	5.58	3.64	7.00	0-10	0-10	-0.38	-1.29
Sexual intercourse worsening ^b^	476 (68.1)	0.91	2.04	0.00	0-10	0-10	2.79	7.60
Dysuria improvement	699	3.73	4.04	2.00	0-10	0-10	0.41	-1.53
Dysuria worsening	699	0.60	1.62	0.00	0-10	0-10	3.45	12.58
Dyschezia improvement	699	4.67	3.87	5.00	0-10	0-10	0.00	-1.58
Dyschezia worsening	699	0.70	1.78	0.00	0-10	0-10	3.17	10.32
**Expected disability (expected PDI)**								
Total sum score	699	15.41	13.77	12.00	0-66	0-70	1.07	0.52
ΔTotal sum score ^c^	699	15.10	14.12	14.00	-36-65	-70-70	0.16	0.34
Total mean score	699	2.20	1.97	1.71	0-9.43	0-10	1.07	0.52
ΔTotal mean score ^c^	699	2.16	2.02	2.00	-5.14-9.29	-10-10	0.16	0.34

*Note. M*, mean; *SD*, standard deviation; *Md*, median; Skew, skewness; Kurt, kurtosis; PDI, Pain Disability Index; NRS, numerical rating scale; PHQ-4, Patient Health Questionnaire, 4-item version; CSQ, Coping Strategies Questionnaire; GEEE, Generic Rating Scale for Treatment Expectations; TEX-Q, Treatment Expectation Questionnaire; Δ, difference score.

^a^ Menstrual bleeding was not applicable for *n*=75.

^b^ Sexual intercourse was not applicable for *n*=223.

^c^ *n*=74 (10.6%) had negative values, that is, expected worsening of disability; *n*=22 (3.1%) had a value of zero, that is, expected no change in disability.

**Figure 2.**
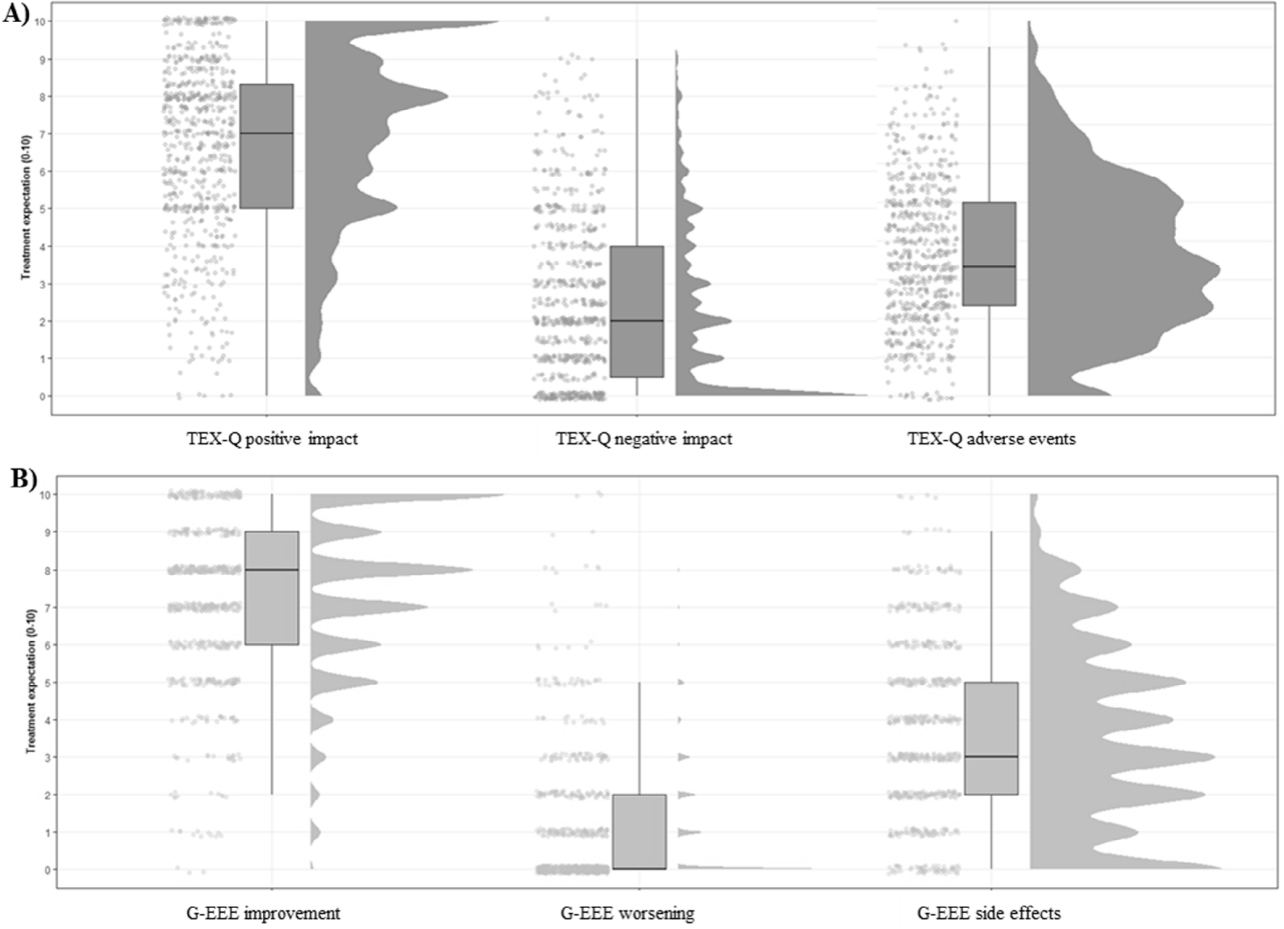
Distributions of treatment expectation measurements using raincloud plots. *Note.* A: TEX-Q subscales: positive impact, negative impact, and adverse events. B: GEEE scale improvement, worsening, and side effects.

In general, participants with suspected endometriosis had rather positive treatment expectations concerning laparoscopy. However, the wide ranges of all measurements, mostly covering the total potential range, indicate pronounced interindividual differences. Positive dimensions (TEX-Q; treatment benefit, positive impact) and items (GEEE, NRS), such as expected improvement, were rated with higher values ( *M*=6.68 to 7.20, *SD*=1.90 to 2.35) and were distributed slightly left-skewed (skewness: -0.84 to -0.64). Negative dimensions (TEX-Q; adverse events, negative impact) and items (GEEE, NRS) such as expected worsening ( *M*=0.92 to 1.09, *SD*=1.79) or side effects ( *M*=3.67 and 3.72, *SD*=1.93 and 2.33) were rated with lower values and distributed clearly right-skewed (0.30 to 3.45). Expected disability (PDI total mean score) after laparoscopy ranged from 0 to 9.43 ( *M*=2.20, *SD*=1.97) and was right-skewed (1.07). Expected change of disability (Δtotal mean score) ranged from -5.14 (medium worsening) to 9.29 (high improvement). Scores tended to be normally distributed based on graphical examination and skewness and kurtosis parameters, whereas the Kolmogorov-Smirnov test was significant ( *D*(699)=.05, *p*< 0.001). The distributions of some exemplary expectation measurements are shown in Figure 2.

### Cluster of treatment expectations

**Figure 3.**
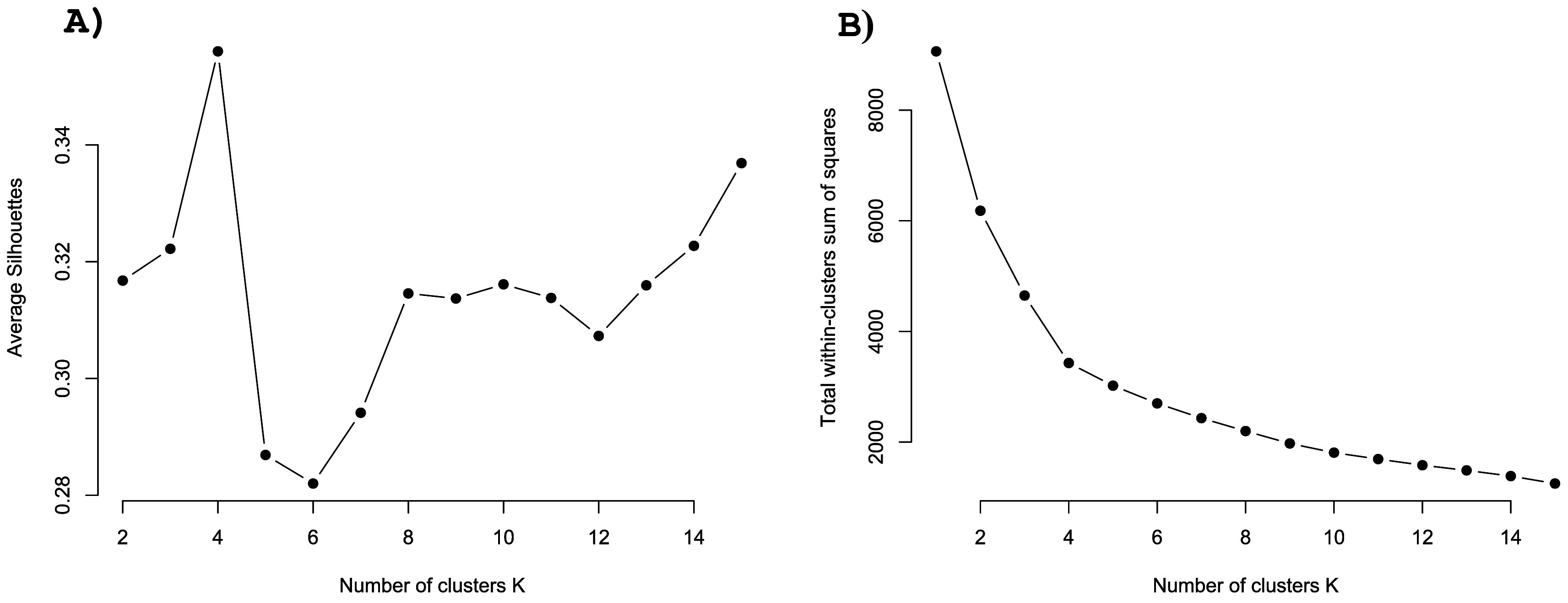
Silhouette and elbow plots for the exploration of possible factors. *Note.* A: Silhouette plot showing the average silhouette score in relation to the number of clusters. B: Elbow plot showing the total within-cluster sum of squares for the number of potential clusters.

**Figure 4.**
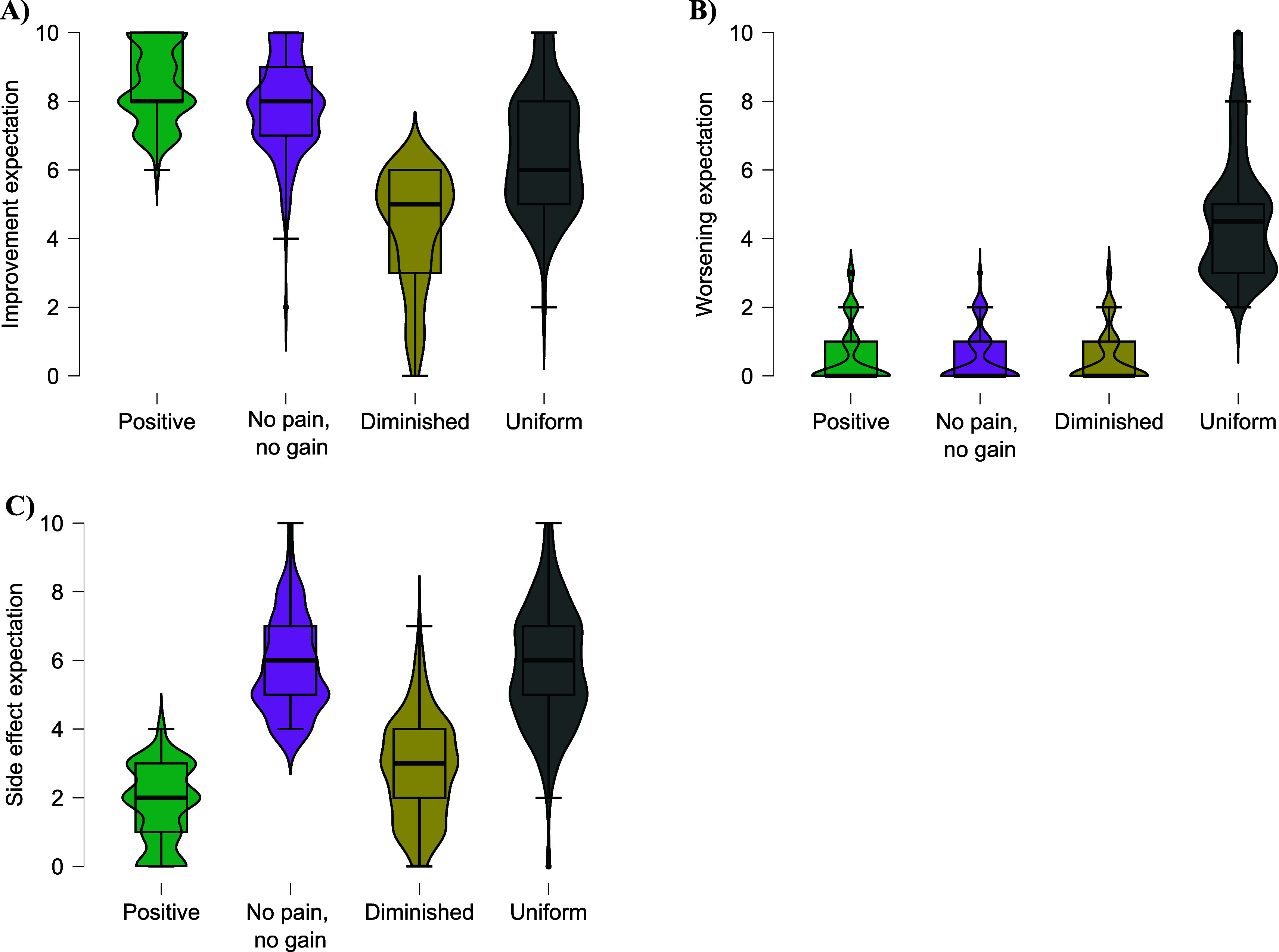
Distribution of the GEEE values for each cluster. *Note.* A: Average GEEE improvement expectation scores for each of the four clusters. B: Average GEEE worsening expectation scores for each of the four clusters. C: Average GEEE side effect expectation scores for each of the four clusters.

Figure 3 shows the resulting elbow and silhouette plots for potential clustering of the three GEEE items (symptom improvement, symptom worsening, and side effects). Both plots suggest *k*=4 is the optimal number of clusters. K-means clustering with *k*=4 resulted in a proportion of explained variance of 62.2%. The details of these clusters are listed in Table 3. Descriptively, the clusters can be described as ‘positive’ (high improvement expectation), ‘no pain, no gain’ (high improvement and side effect expectation), ‘diminished’ (rather low expectations on every dimension), ‘uniform’ (equally high expectations in every direction) (see Figure 4).

**Table 3.** Characteristics of the four identified clusters.

	Cluster ‘positive’ ( *n*=260)	Cluster ‘no pain, no gain’ ( *n*=204)	Cluster ‘diminished’ ( *n*=137)	Cluster ‘uniform’ ( *n*=98)
**Cluster centroids**				
**Generic rating scales (GEEE)** *M* ( *SD*)				
Improvement	8.46 (1.14)	7.85 (1.35)	4.30 (1.67)	6.55 (1.67)
Worsening	0.53 (0.88)	0.47 (0.75)	0.48 (0.81)	4.71 (1.87)
Side effects	1.76 (1.15)	5.84 (1.41)	2.82 (1.45)	5.77 (1.75)
**Demographics per cluster**				
**Mean age** in years ( *SD*)	29.9 (6.6)	30.5 (6.6)	29.2 (6.5)	29.6 (5.7)
***N*** gender (%)				
Female	255 (98.1)	202 (99.0)	132 (96.4)	97 (99.0)
Non-binary	5 (1.9)	2 (1.0)	5 (3.6)	1 (1.0)
***N*** unable to work because of endometriosis-related complaints (%)	21 (8.1)	16 (7.8)	16 (11.7)	23 (23.5)
**Migrant background** *N* (%)				
Yes	53 (25.5)	29 (16.8)	19 (17.0)	28 (34.6)
No	155 (74.5)	144 (83.2)	93 (83.0)	53 (65.4)
**Educational level** *N* (%)				
No school degree	2 (0.8)	0	0	0
In school	3 (1.2)	3 (1.5)	1 (0.7)	1 (1.0)
Lower secondary school	10 (3.8)	1 (0.5)	2 (1.5)	0
Secondary school	73 (28.1)	48 (23.5)	16 (11.7)	33 (33.7)
Higher entrance certificate	88 (33.9)	79 (38.7)	63 (46.0)	44 (34.9)
University degree	84 (32.3)	73 (35.8)	55 (40.1)	20 (20.4)
**Reason for Laparoscopy** *N* (%)				
Complaints	201 (77.3)	156 (76.5)	111 (81.0)	81 (82.7)
Complaints with an unmet wish to have children	59 (22.7)	48 (23.5)	26 (19.0)	17 (17.3)
**Previous experience with laparoscopy** *N* (%)				
Yes	63 (24.2)	38 (18.6)	21 (15.3)	27 (27.6)
No	197 (75.8)	166 (81.4)	116 (84.7)	71 (72.4)
**Disability (PDI)**				
Total mean score ( *SD*)	4.3 (1.8)	4.3 (2.0)	4.0 (2.0)	5.1 (2.0)
**Depressive symptoms (PHQ-4)**				
Total mean score ( *SD*)	2.5 (1.4)	2.5 (1.3)	2.5 (1.5)	2.9 (1.4)
**Anxiety (PHQ-4)**				
Total mean score ( *SD*)	2.4 (1.7)	2.2 (1.5)	2.5 (1.8)	2.9 (1.6)
**Catastrophizing pain (CSQ)**				
Total mean score ( *SD*)	17.3 (7.6)	17.1 (7.7)	16.2 (8.2)	19.8 (7.7)

*Note. SD*, standard deviation; GEEE, Generic Rating Scale for Treatment Expectations; PDI, Pain Disability Index; NRS, numerical rating scale; PHQ-4, Patient Health Questionnaire, 4-item version; CSQ, Coping Strategies Questionnaire.

### Convergent validity: Bivariate associations of all treatment expectation measurements

**Figure 5.**
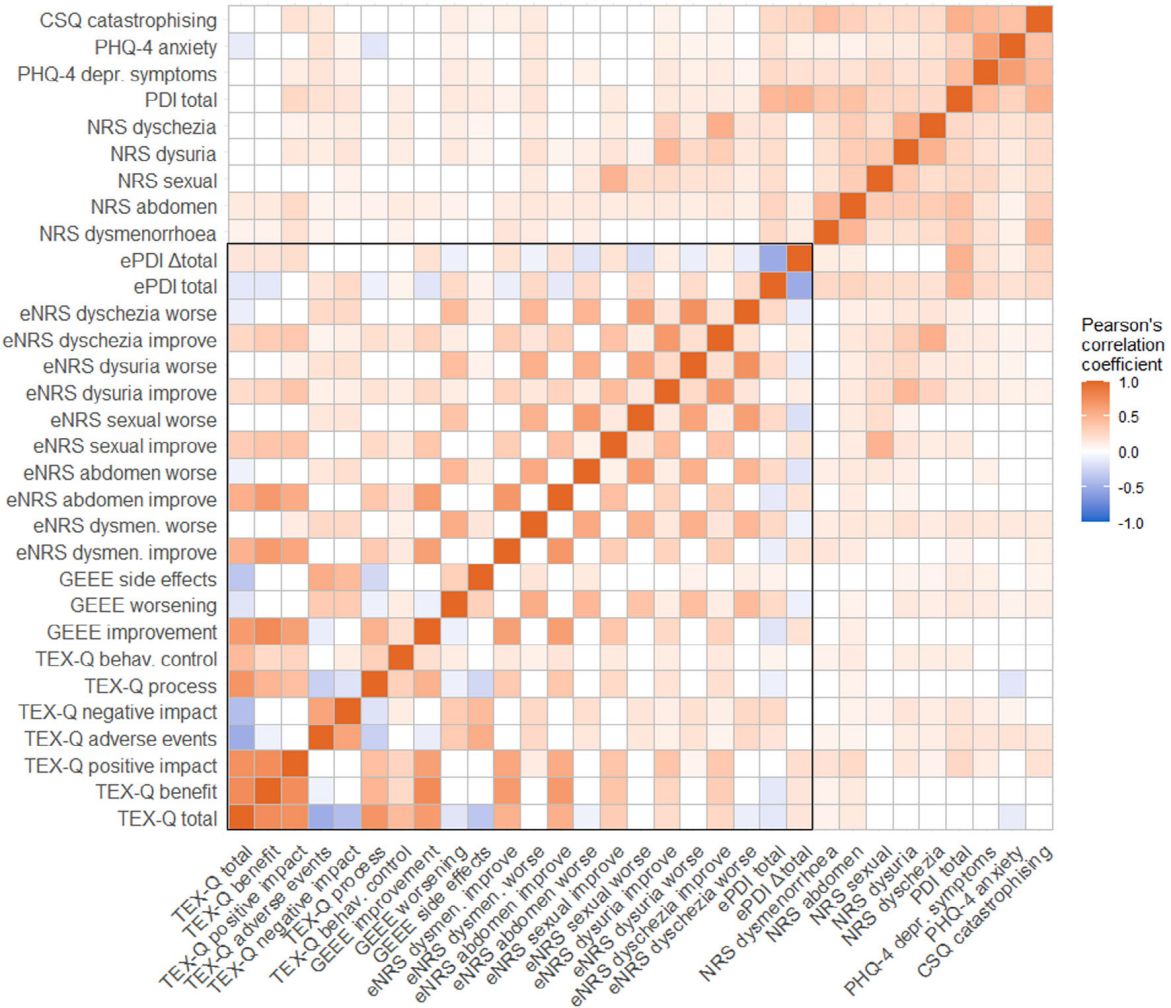
Heatmap representing pearson correlation coefficients between treatment expectation measurements and further psychological constructs. *Note.* Red indicates positive correlations, and blue indicates negative correlations. Non-significant correlations are white. The box highlights the correlations between the treatment expectation measurements (convergent validity). More information on each measurement is provided in the Methods section *Measurements.* The underlying statistics are presented in Table 4. TEX-Q, Treatment Expectation Questionnaire; GEEE, Generic Rating Scale for Treatment Expectations; eNRS, expected numerical rating scales; ePDI, expected Pain Disability Index; Δ, difference score; NRS, numerical rating scales; PDI, Pain Disability Index; PHQ-4, Patient Health Questionnaire, 4-item version; CSQ, Coping Strategies Questionnaire.

All associations of the treatment expectation measurements are presented in Figure 5, with the underlying statistics in Table 4. The dimensions of ‘treatment benefit’ and ‘positive impact’ of the TEX-Q were largely associated with each other ( *r*=.75, *p*<.001), the total score ( *r*=.76, *p*<.001 and *r*=.73, *p*<.001), and with a medium effect size with the expected process ( *r*=.49, *p*<.001 and *r*=.42, *p*<.001, respectively). Negative dimensions (‘adverse events’ and ‘negative impact’) were largely associated with each other ( *r*=.60, *p*<.001) and the total score ( *r*= -.51 to *r*=-40, *p*<.001), but correlations with positive dimensions were non-significant to small. The correlations of the GEEE items were non-significant to small. Participants who expected more worsening from laparoscopy also expected more side effects ( *r*=.31, *p*<.001) and less improvement ( *r*=-.09, *p*=.016), but the effect sizes were small. The expected improvement and side effects of laparoscopy were not associated ( *r*=-.07, *p*=.061). Correlations of expected worsening and improvement of each endometriosis-related symptom (NRS) were non-significant to small ( *r*=.15 to .24, *p*<.001), whereas some expected changes in specific endometriosis-related symptoms were highly associated with the same expected change of other symptoms, for example, improvement of dysmenorrhea and abdominal pain, worsening of dysuria, and dyschezia ( *r*=.69 to.73, *p*<.001). The correlation between expected disability (expected PDI) and change in disability (Δtotal score) was high ( *r*=-.52, *p*<.001); participants who expected more disability after laparoscopy also expected worsening or a smaller reduction in disability.

**Table 4.** Correlation matrix including all treatment expectation measurements and further psychological constructs under analysis ( *N*=699).

	*r*/ *p*																														
Measurement	1.	2.	3.	4.	5.	6.	7.	8.	9.	10.	11.	12.	13.	14.	15.	16.	17.	18.	19.	20.	21.	22.	23.	24.	25.	26.	27.	28.	29.	30.	31.
1. TEX-Q Total score	1																														
2. TEX-Q Treatment benefit	**.762 <.001**	1																													
3. TEX-Q Positive impact	**.731 <.001**	**.748 <.001**	1																												
4. TEX-Q Adverse events	**-.509 <.001**	**-.077 .042**	-.001 .978	1																											
5. TEX-Q Negative impact	**-.401 <.001**	-.022 .566	.070 .065	**-.601 .<.001**	1																										
6. TEX-Q Process	**.706 <.001**	**.491 <.001**	**.419 <.001**	**-.288 <.001**	**-.168 <.001**	1																									
7. TEX-Q Behavioral control	**.448 <.001**	**.243 <.001**	**.284 <.001**	.060 .113	**.117 .002**	**.308 <.001**	1																								
8. GEEE Improvement	**.657 <.001**	**.771 <.001**	**.625 <.001**	**-.109 .004**	**-.043 .255**	**.507 <.001**	**.205 <.001**	1																							
9. GEEE Worsening	**-.164 <.001**	-.057 .134	.063 .096	**.338 <.001**	**.340 <.001**	**-.092 .015**	**.118 <.002**	**-.091 .016**	1																						
10. GEEE Side effects	**.354 <.001**	-.068 .073	-.054 .151	**.544 <.001**	**.449 <.001**	**-.239 <.001**	.014 .702	-.071 .061	**.309 <.001**	1																					
11. eNRS Dysmenorrhoea improvement	**.505 <.001**	**.663 <.001**	**.586 <.001**	**.016 .685**	**.054 .181**	**.352 <.001**	**-.145 <.001**	**.626 <.001**	.013 .744	.003 .942	1																				
12. eNRS Dysmenorrhoea worsening	-.064 .109	.001 .974	**.135 <.001**	**.246 <.001**	**.251 <.001**	-.025 .527	**.095 .018**	**.**011 .775	**.544 <.001**	**.167 <.001**	.065 .106	1																			
13. eNRS Abdomen improvement	**.534 <.001**	**.666 <.001**	**.549 <.001**	-.043 .255	-.016 .668	**.369 <.001**	**.168 <.001**	**.643 <.001**	-.033 .379	-.016 .666	**.691 <.001**	.043 .279	1																		
14. eNRS Abdomen worsening	**-.081 .031**	-.016 .670	.045 .231	**.163 <.001**	**.207 <.001**	-.034 .369	.045 .237	-.030 .431	**.465 <.001**	**.137 <.001**	.021 .597	**.575 <.001**	.014 .710	1																	
15. eNRS Sexual intercourse improvement	**.332 <.001**	**383 <.001**	**.381 <.001**	.013 .775	.042 .366	**.243 <.001**	**.144 .002**	**.365 <.001**	.030 .519	-.002 .972	**.324 <.001**	.094 .051	**.418 <.001**	**.099 .031**	1																
16. eNRS Sexual intercourse worsening	-.063 .169	.013 .772	.053 .253	**.157 <.001**	**.174 <.001**	-.054 .239	.048 .294	-.006 .904	**.393 <.001**	.081 .076	.019 .690	**.509 <.001**	-.006 .890	**.647 <.001**	**.150 .001**	1															
17. eNRS Dysuria improvement	**.232 <.001**	**.270 <.001**	**.381 <.001**	**.099 .009**	**.112 .003**	**.182 <.001**	**.120 .001**	**.255 <.001**	**.120 .001**	.069 .070	**.295 <.001**	**.157 <.001**	**.294 <.001**	**.122 .001**	**.442 <.001**	**.147 .001**	1														
18. eNRS Dysuria worsening	-0.64 .093	.006 .883	**.081 .032**	**.180 <.001**	**.198 <.001**	-.003 .936	.025 .514	.022 .565	**.435 <.001**	**.094 .013**	.056 .163	**.519 <.001**	.015 .701	**.522 <.001**	.087 .058	**.605 <.001**	**.244 <.001**	1													
19. eNRS Dyschezia improvement	**.259 <.001**	**.327 <.001**	**.362 <.001**	**.079 .037**	**.110 .003**	**.206 <.001**	**.145 <.001**	**.290 <.001**	**.124 <.001**	.029 .447	**.316 <.001**	**.166 <.001**	**.321 <.001**	.066 .084	**.399 <.001**	**.119 .009**	**.671 <.001**	**.206 <.001**	1												
20. eNRS Dyschezia worsening	**-.095 .012**	.008 .840	.066 .081	**.245 <.001**	**.254 <.001**	-.032 .392	.071 .060	.000 .996	**.452 <.001**	**.110 .004**	.051 .205	**.470 <.001**	.003 .945	. **495 <.001**	.076 .097	**.628 <.001**	**.170 <.001**	**.732 <.001**	**.183 <.001**	1											
21. ePDI Total mean score	**-.140 <.001**	**-.142 <.001**	.027 .468	**.172 <.001**	**.245 <.001**	**-.087 .022**	**.076 .044**	**-.161 <.0001**	**.238 <.001**	**.093 .013**	**-.095 .017**	**.249 <.001**	**-.126 <.001**	**.236 <.001**	-.052 .255	**.236 <.001**	.032 .405	**.231 <.001**	.007 .850	**.237 <.001**	1										
22. ePDI ΔTotal mean score	**.169 <.001**	**.173 <.001**	**.222 <.001**	.016 .668	-.073 .053	.070 .065	.038 .320	**.195 <.001**	**-.088 .019**	.040 .297	**.182 <.001**	**-.083 .038**	**.188 <.001**	**-.162 <.001**	**.181 <.001**	**-.188 <.001**	**.115 .002**	**-.095 .012**	**.119 .002**	**-.115 .002**	**-.520 <.001**	1									
23. NRS Dysmenorrhoea	**.088 .027**	**.090 .025**	**.191 <.001**	**.084 .036**	.031 .442	.007 .856	**.088 .028**	.061 .127	.064 .112	.045 .265	**.171 <.001**	**.133 <.001**	.051 .199	**.110 .006**	.023 .637	.021 .657	.071 .078	.056 .160	.060 .137	.069 .085	**.248 <.001**	**.119 .003**	1								
24. NRS Abdomen	**.137 <.001**	**.140 <.001**	**.236 <.001**	**.077 .042**	**.090 .018**	**.094 .013**	**.134 <.001**	**.112 .003**	**.089 .019**	.057 .133	**.130 .001**	**.160 <.001**	**.131 <.001**	**.145 <.001**	**.130 .004**	**.144 .002**	**.155 <.001**	**.117 .002**	**.160 <.001**	**.106 .005**	**.283 <.001**	**.120 .001**	**.482 <.001**	1							
25. NRS Sexual intercourse	.024 .609	.031 .497	.080 .081	.068 .139	**.093 .044**	.038 .402	.073 .113	.036 .428	.077 .092	.057 .216	-.004 .928	**.143 .003**	.071 .124	**.149 .001**	**.502 <.001**	**.217 <.001**	**.227 <.001**	**.185 <.001**	**.191 <.001**	**.124 .007**	**.221 <.001**	.031 .504	**.180 <.001**	**.335 <.001**	1						
26. NRS Dysuria	.030 .429	-035 .357	**.161 <.001**	**.121 .001**	**.174 <.001**	.030 .425	**.124 .001**	.067 .075	**.145 <.001**	**.084 .026**	.073 .070	**.194 <.001**	**.084 .026**	**.107 .005**	**.171 <.001**	**.095 .039**	**.473 <.001**	**.245 <.001**	**.319 <.001**	**.160 <.001**	**.218 <.001**	.050 .189	**.202 <.001**	**.332 <.001**	**.343 <.001**	1					
27. NRS Dyschezia	.010 .801	.022 .568	**.086 .023**	**.114 .003**	**.119 .002**	.010 .794	**.124 <.001**	.042 .267	**.106 .005**	**.074 .049**	.068 .092	**.132 <.001**	.036 .345	.034 .373	**.118 .010**	.079 .084	**.297 <.001**	**.143 <.001**	**.527 <.001**	**.175 <.001**	**.189 <.001**	.062 .099	**.221 <.001**	**.334 <.001**	**.219 <.001**	**.507 <.001**	1				
28. PDI total mean score	.034 .372	.035 .352	**.257 <.001**	**.190 <.001**	**.171 <.001**	-.015 .687	**.116 .002**	**.**039 .304	**.149 <.001**	**.135 <.001**	**.090 .024**	**.168 <.001**	.067 .077	.070 .063	**.140 .002**	.039 .395	**.151 <.001**	**.134 <.001**	**.130 <.001**	**.121 .001**	**.471 <.001**	**.509 <.001**	**.374 <.001**	**.409 <.001**	**.258 <.001**	**.271 <.001**	**.255 <.001**	1			
29. Depressive symptoms (PHQ-4)	-.041 .274	.012 .748	**.117 .002**	**.175 <.001**	**.107 .005**	-.056 .138	.005 .900	.006 .884	**.128 <.001**	**.099 .009**	.032 .428	**.158 <.001**	.010 .801	**.100 .008**	.084 .067	.032 .479	**.153 <.001**	**.096 .011**	**.133 <.001**	**.074 .050**	**.237 <.001**	**.190 <.001**	**.187 <.001**	**.178 <.001**	**.252 <.001**	**.194 <.001**	**.210 <.001**	**.435 <.001**	1		
30. Anxiety (PHQ-4)	**-.117 .002**	-.058 .123	.023 .548	**.178 <.001**	**.081 .033**	**-.153 <.001**	-.018 .637	-.061 .107	**.091 .016**	**.**064 .094	-.026 .514	**.152 <.001**	-.063 .096	.047 .212	.001 .987	.018 .702	**.103 .006**	**.078 .039**	**.085 .025**	.041 .280	**.186 <.001**	**.099 .009**	**.101 .011**	**.093 .014**	**.144 .002**	**.136 <.001**	**.181 <.001**	**.290 <.001**	**.637 <.001**	1	
31. Catastrophizing pain (CSQ)	.030 .426	.049 .193	**.193 <.001**	**.161 <.001**	**.067 .077**	-.019 .614	.050 .185	.052 .171	**.111 .003**	**.076 .045**	**.103 .010**	**.143 <.001**	**.030 .433**	.069 .069	.075 .104	.032 .485	**.094 .013**	.067 .076	**.092 .015**	.051 .176	**.236 <.001**	**.273 <.001**	**.417 <.001**	**.297 <.001**	**.219 <.001**	**.232 <.001**	**.227 <.001**	**.520 <.001**	**.454 <.001**	**.402 <.001**	1

*Note.* The significant correlations are indicated in bold. TEX-Q, Treatment Expectation Questionnaire; GEEE, Generic Rating Scale for Treatment Expectations; eNRS, expected numerical rating scales; ePDI, expected Pain Disability Index; Δ, difference score; NRS, numerical rating scales; PDI, Pain Disability Index; PHQ-4, Patient Health Questionnaire, 4-item version; CSQ, Coping Strategies Questionnaire.

The positive dimensions of treatment expectations regarding laparoscopy, the expected process, and the total score of the TEX-Q were highly and positively correlated with the expected improvement in disability as measured by the GEEE ( *r*=.51 to .77, *p*<.001). Medium-sized positive correlations were found between the negative dimensions of the TEX-Q and expected worsening ( *r*=.34, *p*<.001 and *r*=.34, *p*<.001) and expected side effects ( *r*=.45 to.55, *p*<.001), as measured by the GEEE.

Considering the expected improvement and worsening of the severity of endometriosis-related symptoms (NRS), participants who expected more treatment benefit, positive impact from the laparoscopy, and had more positive overall treatment expectations measured by the TEX-Q, also reported more improvement in the severity of symptoms, especially dysmenorrhea ( *r*=.51 to .66, *p*<.001) and abdominal pain ( *r*=.53 to .67, *p*<.001). Negative dimensions of the TEX-Q were significantly correlated with the expected worsening of symptom severity, but the effect sizes were small ( *r*=.16 to.25, *p* <.001). Additionally, the expected improvement and worsening of disability measured by the GEEE were positively correlated with the expected improvement and worsening of the severity of endometriosis-related symptoms (NRS), respectively, ranging from rather high correlations for dysmenorrhea (improvement: *r*=.63 and worsening: *r*=.54, *p*<.001) and abdominal pain ( *r*=.64 and .47, *p*<.001) to rather medium correlations for pain during sexual intercourse ( *r*=.37 and .39, *p*<.001), dysuria ( *r*=.26 and .44, *p*<.001), and dyschezia ( *r*=.29 and.45, *p*<.001).

Considering the expected disability after laparoscopy measured using the expected PDI, all correlations with other treatment expectation measurements were small. Participants who expected lower disability after laparoscopy had more positive treatment expectations overall (r=-.14, *p*<.001) and expected fewer adverse events and negative impact measured by the TEX-Q ( *r*=.17 and.26, *p*<.001). Participants who expected a higher positive change in disability (Δtotal score) by laparoscopy (i.e., improvement of disability) also expected more benefit from treatment, more positive impact, and more positive treatment expectations overall ( *r*=.17 to.22, *p*<.001). No associations were found with the other TEX-Q dimensions. Additionally, the correlations between the expected improvement and worsening of disability measured by the GEEE and expected disability after laparoscopy ( *r*=-.16, *p*<.001 and *r*=.24, *p*<.001) and expected changes in disability ( *r*=.20, *p*<.001 and *r*=-.09, *p*=.02) measured by the PDI were small, although both measurements explicitly referred to endometriosis-related disability. Similarly, almost the same associations regarding direction and effect size were found between expected disability and change in disability measured by the expected PDI and expected improvement and worsening of almost all endometriosis-related symptoms (NRS).

### Discriminant validity: Bivariate associations of treatment expectation measurements and further psychological constructs

Overall, correlations between all treatment expectation measurements and investigated further psychological constructs, that is, preoperative disability, severity of the five most prevalent endometriosis-related symptoms, depressive symptoms, anxiety, and catastrophizing pain, were non-significant to small, with a few exceptions of medium-sized associations. Correlations between treatment expectations measured by the TEX-Q and all psychological constructs were non-significant to small. The most notable correlations were as follows: participants who experienced more preoperative disability and a higher severity of abdominal pain expected a higher positive impact from laparoscopy ( *r*=.26 and .24, *p*<.001). Participants who reported more depressive symptoms and anxiety expected more adverse events ( *r*=.18 and.18, *p*<.001).

The expected improvement, worsening, and side effects measured by the GEEE were not correlated with any of the psychological constructs to a relevant extent ( *r*<.13). Correlations between expected improvement and worsening of the severity of the five most prevalent endometriosis-related symptoms and all further psychological constructs were non-significant to small, despite a few exceptions. Participants who experienced higher pain during sexual intercourse, more severe dysuria, and more severe dyschezia expected considerably more improvement in these specific complaints ( *r*=.47 to .53, *p*<.001). Expected disability after laparoscopy and expected change in disability measured by PDI were highly correlated with preoperative disability. Participants who experienced more preoperative disability expected a higher disability after laparoscopy ( *r*=.47, *p*<.001) and a higher change, that is, improvement of disability by laparoscopy ( *r*=.51, *p*<.001). Additionally, participants who reported more severe endometriosis-related symptoms, more depressive symptoms, and more anxiety expected a higher disability after laparoscopy ( *r*=.19 to .29, *p*<.001). Participants with a stronger tendency to catastrophize pain expected both a higher disability after laparoscopy and a higher chance, that is, improvement of disability from laparoscopy ( *r*=.24 and .27, *p*<.001).

## Discussion

### Summary

People with suspected endometriosis reported rather positive pre-operative treatment expectations concerning laparoscopy, but wide ranges emphasize differences between participants. The negative dimensions indicate major floor effects. As hypothesized, low correlations between the dimensions of treatment expectation measurements indicate that treatment expectation also seems to be a multidimensional construct in people with suspected endometriosis. Thus, the calculation of the total sum scores might not be indicated or proven on a case-by-case basis. No association was found between the expected improvement and the side effects of laparoscopy in all patients. Four distinct clusters described as ‘positive’, ‘no pain, no gain’, ‘diminished’, and ‘uniform’ were identified. In line with our second hypothesis, the treatment expectation measurements TEX-Q and GEEE correlated higher with each other in terms of convergent validity compared to other psychological measurements assessed in terms of discriminant validity. This applies to the equivalent dimensions of both. Additionally, the expected improvement and worsening of disability (GEEE) and the severity of the five most prevalent endometriosis-related symptoms (NRS) led to medium-to-high positive associations of equivalent dimensions. Both scales used similar wording but focused on different constructs. Contrary to our second hypothesis of convergent validity, correlations of expected disability after laparoscopy measured by the expected PDI and most other measurements of treatment expectations were non-significant to small. However, none of the investigated measurements of treatment expectation correlated to such an extent that they measured exactly the same construct.

### Comparison with existing literature

Treatment expectations measured with the TEX-Q of people with suspected endometriosis undergoing laparoscopy were slightly lower than those of the validation sample ^34^ for the total score and all dimensions, despite equal scores for the expected ‘negative impact’ of treatment. This contradicts the findings of more positive treatment expectations in surgical treatment samples undergoing endocrine or bariatric surgery than in psychosomatic samples. ^34^ Intercorrelations of dimensions were similar in both studies despite of higher correlations between the positive dimensions ‘treatment benefit’ and ‘positive impact’ ( *r*=.75 in our sample vs. *r*=.48 in the validation sample ^34^) and ‘positive impact‘and ‘process‘( *r*=.42 vs. *r*=.15). Moreover, the dimension ‘behavioral control’ was significantly correlated to ‘treatment benefit’ ( *r*=.24), ‘negative impact’ ( *r*=.12), and ‘process’ ( *r*=.31) in people with suspected endometriosis but not in the validation sample. Correlations of the TEX-Q and other psychological constructs were small to non-significant in our study and the validation study, ^34^ indicating discriminant validity. Interestingly, participants in our study who reported more depressive symptoms and anxiety expected more adverse events. More unfavorable expectations, that is, lower expected treatment benefit, higher negative impact, and a worse expected process, were also reported in patients with higher depressive and anxiety symptoms in the TEX-Q validation sample. ^34^ For the other assessed treatment expectation measurements, no validation data are currently available.

In line with Shedden-Mora et al. (2023) ^34^ and Younger et al. (2012), ^30^ our findings support the multidimensionality of the construct of treatment expectations: the positive and negative dimensions of treatment expectations were not correlated in these three studies. Overall, the expected side effects of laparoscopy and its improvement were not associated with the results of patients undergoing psychotherapy. ^46^ This contradicts the ‘no pain, no gain’ assumption.

So far, research on psychometric properties of measuring treatment expectations ^30^^,^^34^ has used heterogeneous samples including patients undergoing different surgeries, pain, psychosomatic in- or outpatient, or cancer treatment. Differences between patient groups and within patient groups could hardly be detected, but seem promising. Our cluster analysis revealed four distinct clusters in patients with suspected endometriosis that differed in terms of their treatment expectation pattern. These clusters can be described as follows. The first cluster showed a strong positive expectation, followed by no worsening or side effect expectations. For the second cluster, the ‘no pain, no gain’ assumption seems to be appropriate; these patients expected high side effects of laparoscopy and high improvement. In the third cluster, we found an overall low level of expectations for any category. The fourth cluster had a rather uniform distribution of expectations, with all expectations being of similar strength. Notably, only patients in this cluster patients also expected worsening. The largest cluster was the first with an improvement in expectation dominance, supporting the rather high expectations found in this sample.

### Strengths and limitations

Our findings are based on data from a large sample of people with suspected endometriosis facing laparoscopy in a specialized center for surgical endoscopy and endometriosis, indicating high ecological validity. We assessed four measurements for treatment expectation (TEX-Q, the three items assessing expectations of the GEEE, expected NRS, and expected Pain Disability Index) simultaneously. Consequently, we were able to compare different dimensions, comprehensive questionnaires to single items, and treatment expectation measurements (TEX-Q and GEEE) to established symptom and disability scales (NRS assessing pain and PDI) adapted to the context of expectations. To our knowledge, this is the first study to investigate the treatment expectations in people with suspected endometriosis. Endometriosis is a common chronic disease that is largely underexplored and burdensome, and satisfactory treatment options are not available for many patients.

However, the timeframe of the assessed treatment expectations was not well defined in this study. People with suspected endometriosis might have different expectations for the short-, medium-, and long-term outcomes after laparoscopy. We were unable to quantify these potential differences. The order of the measurement instruments was the same for all participants, and sequence effects were possible. To enhance comprehensibility, the survey started with items referring to the past, then to the present, and finally to the future. Consequently, expected disability (expected PDI) and severity of symptoms (expected NRS) were not assessed directly after the preoperative disability and severity of symptoms. Some patients may have forgotten their reference values, leading to imprecise ratings. Finally, some anchors (i.e., the greatest improvement/worsening imaginable) might be understood unequally by participants with suspected endometriosis. Qualitative data from the embedded study module of this cohort study ^33^ may provide further clarifications.

### Implications for practice and research

Women with suspected endometriosis are highly burdened and report rather positive expectations concerning laparoscopy, even though laparoscopy is associated with only short-term symptom improvement, ^15^ and 20–30% do not respond satisfactorily to this treatment. ^16^^,^^17^ Future studies should investigate whether more positive expectations lead to better treatment outcomes, disappointment due to overly optimistic expectations or do not have any impact. Based on knowledge of other medical conditions ^1^ and surgical treatments, ^4^^,^^5^ positive yet realistic expectations (placebo effect) and reduced negative expectations (nocebo effect) may improve treatment outcomes after laparoscopy. From a clinical perspective it is also interesting what factors shape patients’ expectations. This study used data from the baseline assessment of a mixed-method clinical observational study. ^33^ Structured content analysis of qualitative interviews and longitudinal analyses are in progress to provide new insights to this research gap. For other patient groups it is known that prior information shape patients‘expectations: This includes verbal information (e.g., preoperative information), previous experiences (e.g., personal history of patients), observational learning (e.g., experiences and recommendations of close social contacts or media), and contextual factors (e.g., doctor-patient-relationship). ^47^^–^^49^

Our results emphasize that treatment expectations should be measured both multidimensionally and comprehensively. The expected improvement and worsening are not the anchors of a single scale. The dimension and linguistic formulation seem more important than the exactly chosen construct, that is, the disability or severity of symptoms. None of the included measurements comprised different time frames. In future research, it is important to define time frames for treatment expectation measurements, especially for patients undergoing surgery, such as laparoscopy. To generalize our findings across patient groups, provide specific recommendations for patients with symptoms of endometriosis, and classify expectations in terms of being real, that is, plausible or ideal ^23^^,^^25^ future qualitative and quantitative longitudinal research is needed. The four clusters of expectations need to be replicated and confirmed because of their high relevance to clinical implications. Individualized interventions could target at expectation manipulation such as focusing on i) side effect management in the ‘no pain, no gain’ and ‘uniform’ expectation clusters, ii) increased positive expectations fostering the placebo effect in the ‘diminished’ cluster, iii) decreased negative expectations reducing the nocebo effect and/or appropriate coping mechanisms, and iv) positive but yet realistic expectations in the ‘positive’ cluster.

As we found differences between the measurements of treatment expectations and dimensions, it is very interesting whether clinical outcomes may also differ depending on the chosen measurement. In the past, patients’ expectations were often assessed heterogeneously without conceptual standardization and psychometric evaluation. ^23^ Consequently, the selection of measurements should be carefully considered and either tailored to the respective theory-based primary outcome or defined in a general way to be applicable across treatments.

### Ethical approval

Ethical approval was obtained from Psychotherapeutenkammer Hamburg, Germany (ROXWELL-2021-HH, 25 ^th^ of June 2021). This trial was registered at ClinicalTrials.gov (ID: NCT05019612).

## Data Availability

PsychArchives: Dataset for: Assessment of treatment expectations in women with suspected endometriosis: A psychometric analysis, https://doi.org/10.23668/psycharchives.13585. ^45^ Data are available under the terms of the Creative Commons Attribution 4.0 International license (CC-BY-SA 4.0).
