# Supplementary material for: Assessment of treatment expectations in people with suspected endometriosis: A psychometric analysis
Source: F1000Res. 2024 Sep 9;13:174. Originally published 2024 Mar 11. [Version 2] doi: 10.12688/f1000research.145377.2 (PMC11425038; doi:10.12688/f1000research.145377.2)
Supplement: Peer review history

# Reviewer report: Reviewer response for version 2

**DOI:** 10.5256/f1000research.170250.r323254

**Published:** 2024-10-04

**Authors:** Sonia R Grover

**Affiliations:** 1 Royal Children's Hospital, Murdoch Children's Research Institute, The University of Melbourne, Melbourne, Victoria, Australia

Thankyou for the opportunity to review this paper which is highly relevant. As a gynaecologist and pain medicine specialist I am troubled by the expression ‘suspected endometriosis’. 

 Many studies report that only 50% of women undergoing a laparoscopy for pain will have endometriosis found. With only a 50/50 chance that endometriosis will be present, then the expression has questionable validity. The expression ‘suspected endometriosis’ may be appropriate where imaging has clearly demonstrated endometriomas or deep infiltrating endometriosis, but in Australia many laparoscopies are done for period and pelvic pain where at least ultrasound imaging has been undertaken and has been reported as basically normal. The women here would still undergo a laparoscopy having been convinced by social media, public endometriosis awareness campaigns and probably their primary care specialist as well as their gynaecologist that there is likely endometriosis present to explain their pain. I am aware that the pressure to ‘make a diagnosis’ and papers on the ‘delay to diagnosis’ have come out of Germany so I suspect there is enough similarity between our countries and attitudes to endometriosis.  Yet we know that 50% of the people with period and pelvic pain will not have endometriosis found and they are often more frustrated and confused and invalidated following their laparoscopy.

 Thus I agree with the previous reviewers comment that the cohort should be divided into those undergoing laparoscopy for pain and those undergoing laparoscopy where imaging has demonstrated an identifiable problem as expectation and level of suspicion will be substantially different – both at the clinician level but also thus to the patient. Efforts for others to replicate your study would likewise need to define the presentation of their population as this will influence expectations. Furthermore – it is important to know if this is a first laparoscopy or repeat laparoscopy , as this will also impact the persons response to  the questions around their expectations – and this should be noted.

 There is also a pressing need for studies to be undertaken exploring the outcome of women after their laparoscopy – in particular those with a “negative” laparoscopy – where endometriosis is not found – as there is a need to counter the significant media around the reported importance of endometriosis particularly in the presence of pain, when the evidence is far from clear that this is a clear relationship ( given that people who menstruate with no pain have an almost 50% rate of endometriosis).

 I do hope your work will follow this cohort for >12 months and reassess as well as re-analyse this preliminary data. A question that would then also be relevant is to know whether the patients had been forewarned that the laparoscopy might be negative.

Is the work clearly and accurately presented and does it cite the current literature?

Partly

If applicable, is the statistical analysis and its interpretation appropriate?

I cannot comment. A qualified statistician is required.

Are all the source data underlying the results available to ensure full reproducibility?

Yes

Is the study design appropriate and is the work technically sound?

Yes

Are the conclusions drawn adequately supported by the results?

Partly

Are sufficient details of methods and analysis provided to allow replication by others?

Partly

Reviewer Expertise:

Gynaecologist and a Pain Medicine Specialist – with research interest in period and pelvic pain

I confirm that I have read this submission and believe that I have an appropriate level of expertise to confirm that it is of an acceptable scientific standard, however I have significant reservations, as outlined above.

# Reviewer report: Reviewer response for version 2

**DOI:** 10.5256/f1000research.170250.r321848

**Published:** 2024-09-25

**Authors:** Claire E Lunde

**Affiliations:** 1 Lunde Consulting, LLC, Washington, DC, USA

Thank you to the authors for thoroughly addressing my questions and concerns, and for your valuable contributions to advancing the understanding of endometriosis. The manuscript is scientifically sound in its current form.

Is the work clearly and accurately presented and does it cite the current literature?

Yes

If applicable, is the statistical analysis and its interpretation appropriate?

I cannot comment. A qualified statistician is required.

Are all the source data underlying the results available to ensure full reproducibility?

Yes

Is the study design appropriate and is the work technically sound?

Yes

Are the conclusions drawn adequately supported by the results?

Partly

Are sufficient details of methods and analysis provided to allow replication by others?

Partly

Reviewer Expertise:

Public Health, Behavioral Sciences, Neuroscience, Endometriosis, and Female-Pain,

I confirm that I have read this submission and believe that I have an appropriate level of expertise to confirm that it is of an acceptable scientific standard.

# Reviewer report: Reviewer response for version 2

**DOI:** 10.5256/f1000research.170250.r325045

**Published:** 2024-09-19

**Authors:** D Raimondo

**Affiliations:** 1 Division of Gynaecology and Human Reproduction Physiopathology, S. ORSOLA, Universitaria di Bologna, Bologna, Italy

The study aims to psychometrically analyze and compare different treatment expectation measurements in a sample of women with suspected endometriosis. Additionally, it seeks to describe and exploratively cluster treatment expectations in this patient group.

 Women with suspected endometriosis reported positive expectations concerning laparoscopy, but wide variations indicated significant interindividual differences.  The identified clusters provide initial indications for individualized interventions targeting expectation manipulation.

 Please discuss on side events of endometriosis surgery providing to the readers some examples and their burden (Ref -1,2)

Is the work clearly and accurately presented and does it cite the current literature?

Yes

If applicable, is the statistical analysis and its interpretation appropriate?

Yes

Are all the source data underlying the results available to ensure full reproducibility?

Yes

Is the study design appropriate and is the work technically sound?

Yes

Are the conclusions drawn adequately supported by the results?

Yes

Are sufficient details of methods and analysis provided to allow replication by others?

Yes

Reviewer Expertise:

endometriosis

I confirm that I have read this submission and believe that I have an appropriate level of expertise to confirm that it is of an acceptable scientific standard, however I have significant reservations, as outlined above.

# Reviewer report: Reviewer response for version 1

**DOI:** 10.5256/f1000research.159318.r293041

**Published:** 2024-07-10

**Authors:** Claire E Lunde

**Affiliations:** 1 Lunde Consulting, LLC, Washington, DC, USA

**Summary.**

 Thank you for the opportunity to review the manuscript entitled “Assessment of treatment expectations in women with suspected endometriosis: A psychometric analysis” submitted to F1000Research. The well-written manuscript compared different but related measurements for assessing treatment expectations and thoroughly explored treatment expectations in a large sample of adult patients presenting for endometriosis-suspected laparoscopic surgery. The overarching aims of the current study were to:

 (1) Psychometrically analyze and compare four treatment expectation measurements: TEX-Q, GEEE, NRS, & PDI.

 (2) Describe treatment expectations and potentially influential factors (e.g., depression, anxiety, pain catastrophizing) in adult patients with suspected endometriosis.

 I recommend adding a third aim: (3) Compare treatment expectation results to those of similar conditions and the general population.

 The findings of this study are significant as they provide new insights into treatment expectations for a wide range of patients with endometriosis. These insights could have important implications for personalized patient care and improved treatment outcomes.

**General.**

 The study aims, and hypotheses could be more consistent throughout the manuscript. It would be helpful to the reader if the flow were consistent for each section (e.g., study Aim 1: compare treatment measures; Study Aim 2: describe treatment expectations). New topics and variables are introduced throughout the manuscript. For example, depression, pain catastrophizing, anxiety, and previous experience with laparoscopy aren’t really mentioned in the abstract or introduction. The same is true when comparing treatment expectations to other conditions and the general population. To improve the manuscript’s readability and coherence, the authors could consider providing a clear outline of the manuscript’s structure in the introduction.

 It would be helpful if the authors explicitly stated that only the variables pre-surgery were analyzed. Thus, the effects in clinical practice must be inferred from other conditions researched, such as acute/chronic pain, surgeries, breast cancer, and psychiatric disorders (as listed in the introduction). It would be helpful for the reader if the authors listed these specific clinical effects and recommendations for future researchers/clinicians.

 Since 13 individuals identified as non-binary, I recommend that the title and manuscript use people or patients with endometriosis rather than women with endometriosis.

**Abstract.**

 The abstract provides a concise overview of the study, but it could be improved by including all measures given – depression, anxiety, and pain catastrophizing measures are not listed. In the results section of the abstract, the abbreviation PVE should be spelled out. The authors should also include results for both aims. The average age and age range for the sample should be listed. Lastly, the authors should provide a brief statement on the implications of their findings, which will help readers understand the significance of the study.

**Keywords.**

 Recommend adding NRS and PDI to the keywords.

**Introduction.**

 Please provide a citation for the following sentence on page 4: *The five most prevalent symptoms of endometriosis are dysmenorrhea, abdominal pain, dyspareunia, dyschezia, and dysuria.*

 Recommend describing the often-comorbid relationship between anxiety, depression, pain catastrophizing, and endometriosis-related pain & disability.

**Methods.**

 Please clarify whether the study recruited participants exclusively aged 18 and older or included a broader age range. Were there any specific criteria related to menopausal status? Consider including details about participants’ family history, especially if close relatives (e.g., mother, sister, aunt) had endometriosis or underwent laparoscopic surgery. These social factors may impact expectations. Was this the participant’s first surgery or significant medical treatment? Were there any previous trauma experiences or current chronic stress? If possible, include the duration of endometriosis symptoms, age at diagnosis, treatments attempted, and any prior endocrine therapy. Many patients use social networks or endometriosis support groups for information. Recommend acknowledging the influence of these sources and other anecdotal evidence. Include how well-informed participants were about endometriosis and the possible surgical outcomes. Recommend adding a table describing and comparing the four treatment expectation measures.

**Results.**

 Figure 1 reports n=23 unmet wish to have children, and Table 1 reports n=150 unmet wish to have children -- please clarify. If possible, horizontal tables would be easier to read. Previous experience with laparoscopy was reported in the cluster results but not the descriptive results. Table 2 reports: *N female (divers)* – typo?

**Discussion.**

 What specific individual patient metrics/characteristics do the authors suggest should be investigated in future studies? Similar to the abstract, a more in-depth analysis of the conclusions and implications should be included. What should a clinical population do with the current findings, and what implications should be considered for future studies? This is a fantastic study – specific steps on “what next” would be incredibly beneficial to understanding tailored treatment approaches for patients with endometriosis.

Is the work clearly and accurately presented and does it cite the current literature?

Yes

If applicable, is the statistical analysis and its interpretation appropriate?

I cannot comment. A qualified statistician is required.

Are all the source data underlying the results available to ensure full reproducibility?

Yes

Is the study design appropriate and is the work technically sound?

Yes

Are the conclusions drawn adequately supported by the results?

Partly

Are sufficient details of methods and analysis provided to allow replication by others?

Partly

Reviewer Expertise:

Public Health, Behavioral Sciences, Neuroscience, Endometriosis, and Female-Pain,

I confirm that I have read this submission and believe that I have an appropriate level of expertise to confirm that it is of an acceptable scientific standard, however I have significant reservations, as outlined above.

# Response

**Published:** 2024-08-09

**Authors:** Ann-Katrin Meyrose

**Affiliations:** Clinical Psychology and Psych, Helmut-Schmidt-University / University of the Armed Forces Hamburg, Germany

**Numbered Reviewer’s comments -->** ***Authors’ responses in italic (*** **Change in manuscript new word version in parentheses)**

 1. Summary.

 Thank you for the opportunity to review the manuscript entitled “Assessment of treatment expectations in women with suspected endometriosis: A psychometric analysis” submitted to F1000Research. The well-written manuscript compared different but related measurements for assessing treatment expectations and thoroughly explored treatment expectations in a large sample of adult patients presenting for endometriosis-suspected laparoscopic surgery. The overarching aims of the current study were to:

 (1) Psychometrically analyze and compare four treatment expectation measurements: TEX-Q, GEEE, NRS, & PDI.

 (2) Describe treatment expectations and potentially influential factors (e.g., depression, anxiety, pain catastrophizing) in adult patients with suspected endometriosis.

 I recommend adding a third aim: (3) Compare treatment expectation results to those of similar conditions and the general population.

 The findings of this study are significant as they provide new insights into treatment expectations for a wide range of patients with endometriosis. These insights could have important implications for personalized patient care and improved treatment outcomes.

*--> Thank you very much for your time and valuable feedback, especially from the clinical perspective. The manuscript has a methodological focus on expectations, but we agree that the clinical perspective is also very interesting and should be considered more deeply.*

**2. General.**

 The study aims, and hypotheses could be more consistent throughout the manuscript. It would be helpful to the reader if the flow were consistent for each section (e.g., study Aim 1: compare treatment measures; Study Aim 2: describe treatment expectations).

 New topics and variables are introduced throughout the manuscript. For example, depression, pain catastrophizing, anxiety, and previous experience with laparoscopy aren’t really mentioned in the abstract or introduction. The same is true when comparing treatment expectations to other conditions and the general population. To improve the manuscript’s readability and coherence, the authors could consider providing a clear outline of the manuscript’s structure in the introduction.

*--> Thank you for this comment. We restructured the paragraph describing our study aims for a better flow and consistency throughout the manuscript. The first aim is now to describe treatment expectations (including cluster), the second aim is comparing expectation measures (including convergent and discriminant validity) (*Introduction, p. 5 (Aims and hypotheses). *We checked the order in the whole manuscript: *

- *The first paragraph of the introduction focuses on expectations in people with (suspected) endometriosis, the second on measurement aspects. *
- *In the results section, all results referring to the first study aim are presented first (describing expectations including cluster), all results referring to the second aim (comparing measures, especially measurement validity) afterwards.*
- *Same in the summary of the discussion and implications for practice and research*

*Yet, we did not describe variables such as depression, pain catastrophizing and anxiety in the introduction, because we use those only to interpret the discriminant validity of expectation measures (please see second hypothesis) and to give a brief descriptive overview of the sample under analysis. In this paper, we do not focus on relationships of those constructs from a clinical perspective. Nevertheless, we assessed those constructs in our study, because depression, pain catastrophizing and anxiety seem to be associated with persistent complaints after laparoscopy. To make that clear, we added a sentence to the introduction (and three references).*

*Because readers (e.g., reviewer 1) seem to be interested in clinical data, we added what we assessed to describe the sample more deeply. * (Introduction, first paragraph, p. 5, Reference list)

 3. It would be helpful if the authors explicitly stated that only the variables pre-surgery were analyzed. Thus, the effects in clinical practice must be inferred from other conditions researched, such as acute/chronic pain, surgeries, breast cancer, and psychiatric disorders (as listed in the introduction).

*--> Thank you for this note. We added the information that only pre-operative variables were considered to different parts of the manuscript. (*Abstract – Methods p. 4; Aims and Hypotheses p.5; Methods, first sentence p.6; Discussion – Summary, first sentence p.26)

 4. It would be helpful for the reader if the authors listed these specific clinical effects and recommendations for future researchers/clinicians.

*--> We added information on effect sizes for pain, depression, analgesic treatments, and postsurgery quality of life. (*Introduction, second sentence, p.4)

 5. Since 13 individuals identified as non-binary, I recommend that the title and manuscript use people or patients with endometriosis rather than women with endometriosis.

*--> We totally agree, thanks for this important recommendation. We used people and participants instead of women. (*whole manuscript)

**6. Abstract.**

 The abstract provides a concise overview of the study, but it could be improved by including all measures given – depression, anxiety, and pain catastrophizing measures are not listed. In the results section of the abstract, the abbreviation PVE should be spelled out. The authors should also include results for both aims. The average age and age range for the sample should be listed. Lastly, the authors should provide a brief statement on the implications of their findings, which will help readers understand the significance of the study.

*--> Thank you for these recommendations. Due to the limited space of 300 words, we deleted the sentence about the PVE to include the age range. We restructured the order of sentences to clarify our research aims and corresponding results and conclusions (see also general comment above). We added a second recommendation for future studies and rephrased the first to clarify. Because of this we deleted some words in the abstract to comply with the word limit.*

*After discussion, we decided not to include all measures in the abstract due to limited space. The expectation measurement is the focus of the manuscript, the measures depressive symptoms, anxiety and pain catastrophizing were primarily used to analyse discriminant validity. (*Abstract, p.4)

**7. Keywords.**

 Recommend adding NRS and PDI to the keywords.

*--> We added both to the keywords. (*keywords)

**8. Introduction.**

 Please provide a citation for the following sentence on page 4: *The five most prevalent symptoms of endometriosis are dysmenorrhea, abdominal pain, dyspareunia, dyschezia, and dysuria.*

*--> We added a citation for this sentence (Chiantera V, Abesadze E, Mechsner S. How to Understand the Complexity of Endometriosis-Related Pain. Journal of Endometriosis and Pelvic Pain Disorders 2017; 9(1):30–8. DOI:10.5301/je.5000271.). (*Introduction, first paragraph, p. 5, reference list)

 9. Recommend describing the often-comorbid relationship between anxiety, depression, pain catastrophizing, and endometriosis-related pain & disability.

*--> We assessed depression, pain catastrophizing and anxiety in our longitudinal study, because those constructs seem to be associated with persistent complaints after laparoscopy. To make that clear, we added a sentence to the introduction (and three references). (Introduction, first paragraph, p. 5, Reference list)*

**10. Methods.**

 Please clarify whether the study recruited participants exclusively aged 18 and older or included a broader age range. Were there any specific criteria related to menopausal status?

*--> We recruited and included only participants aged 18 and older. This was checked during the online screening process. One interested person was <18 and excluded (please see Figure 1: Flow chart). Due to the sensitive data assessed in our longitudinal study we decided to focus on adult participants. We rephrased “adult people” into “people aged 18 and older” to clarify. We defined no specific criteria related to menopausal status. (*Methods section, Participants, first sentences, p.6)

 11. Consider including details about participants’ family history, especially if close relatives (e.g., mother, sister, aunt) had endometriosis or underwent laparoscopic surgery. These social factors may impact expectations. Was this the participant’s first surgery or significant medical treatment? Were there any previous trauma experiences or current chronic stress?

 If possible, include the duration of endometriosis symptoms, age at diagnosis, treatments attempted, and any prior endocrine therapy. Many patients use social networks or endometriosis support groups for information. Recommend acknowledging the influence of these sources and other anecdotal evidence. Include how well-informed participants were about endometriosis and the possible surgical outcomes.

*--> Thank you very much for these interesting recommendations from a clinical perspective. Due to the limited space in the preoperative online questionnaire and the lack of previous evidence focusing on the expectations of patients with endometriosis, we decided to concentrate on assessing expectations through various measurements as well as disability, complaints, anxiety, depression, sociodemographics, and some medical characteristics. We added information about what is known to shape expectations for other patient group to the discussion section.*

*We added what we assessed: the self-reported duration of endometriosis symptoms, previous experience with laparoscopy (yes/no), current endocrine therapy (yes/no), and whether complaints are dependent on menstrual bleeding.*

*To the best of our knowledge, our study is the first to investigate expectations in patients with endometriosis. To gain a deeper understanding of these expectations, we also conducted qualitative interviews before and after laparoscopy in an additional study module. The corresponding manuscript is currently under review, and if possible, we would be pleased to link it to this manuscript. As you suggested, patients reported that personal history, prior treatment experiences, pre-operative information and knowledge, stress, and social support are important factors for them (among others). (* *Measurements, last paragraph, p.8,* *Results, second paragraph, p.9, Table 2,* *Discussion section, page 29)*

 12. Recommend adding a table describing and comparing the four treatment expectation measures.

*--> That is a great idea to enhance comprehensibility. We added such a Table to the method section. (*See new Table 1)

**13. Results.**

 Figure 1 reports n=23 unmet wish to have children, and Table 1 reports n=150 unmet wish to have children -- please clarify.

*--> N=23 reported that the unmet wish to have children is the only reason for laparoscopy, N=150 that complaints and an unmet wish to have children is the reason. Those 23 people displayed in the flow diagram were interested to participate but excluded during our screening process. To be included in our longitudinal study endometriosis-related complaints have to be present because we are interested in persisting complaints despite successful laparoscopy.*

*As this point seems misleading, we added this aspect describing our target population in the method section. (*Methods section, Participants, p.6)

 14. If possible, horizontal tables would be easier to read.****

*--> We ask the editorial office, whether horizontal tables are possible.*

 15. Previous experience with laparoscopy was reported in the cluster results but not the descriptive results.

*--> Thank you, we added this information to the table describing the total sample. (*Table 3)

 16. Table 2 reports: *N female (divers)* – typo?

*--> Thanks, that was misleading. We changed the format using N(%) and three lines. (*Table 4)

**17. Discussion.**

 What specific individual patient metrics/characteristics do the authors suggest should be investigated in future studies? Similar to the abstract, a more in-depth analysis of the conclusions and implications should be included. What should a clinical population do with the current findings, and what implications should be considered for future studies? This is a fantastic study – specific steps on “what next” would be incredibly beneficial to understanding tailored treatment approaches for patients with endometriosis.

*--> We added some more recommendations for future studies but are careful with clinical implications. From our point of view we need some more data to develop tailored treatment approaches for patients with endometriosis, i.e.:*

- *Longitudinal data to answer the question whether more positive expectations lead to better treatment outcomes or disappointment due to overly optimistic expectations (or whether expectations do not have an impact on post-operative outcomes - against our hypotheses)*
- *qualitative interviews to understand expectation of people with endometriosis in more depth and knowledge about factors shaping expectations in this patient group*

*As we are currently analyzing the data above and this manuscript ‘only’ uses pre-operative baseline data of our mixed-method clinical observational study (over a 12-month period, plus two additional study modules) we are we are confident to provide more findings in the future to develop a tailored treatment approach for patients with endometriosis. That would be the overarching objective of our research project. (*Discussion section, Implications for practice and research, first paragraph, p. 28/29)

# Reviewer report: Reviewer response for version 1

**DOI:** 10.5256/f1000research.159318.r268828

**Published:** 2024-07-01

**Authors:** Michel Canis

**Affiliations:** 1 CHU Clermont-Ferrand, Department of Gynecologic Surgery, Université Clermont Auvergne, Clermont-Ferrand, France

This is large study about patients expectation before a laparoscopic treatment of endometriosis. This point is very interesting. The methods used appear appropriate, but I am not a specialist of treatment expectations studies.

 I would suggest to include data about the anatomical severity of the disease. A global approach of endometriosis is in my mind always somewhat misleading. Treatment expectations from a laparoscopic procedure are likely very different from a patient who has a mechanical infertility related to adhesions to another one who has no disease at ultrasound and complains of severe pain.

 Data about pre operative information and particularly about the risks of complications would be essential to understand these expectation. A patient "afraid of a bowel fistula" may have different expectation that patients with expected minimal or moderate disease based on preoperative imaging.

 When dealing with pain treatment these data would be very interesting. I would also recommend to collect data about the personal history of these patients, trauma and violence may influence the trust in treatment and the results of this very interesting study. I think that most of these data are available to the authors and could be added to the paper.

Is the work clearly and accurately presented and does it cite the current literature?

Yes

If applicable, is the statistical analysis and its interpretation appropriate?

I cannot comment. A qualified statistician is required.

Are all the source data underlying the results available to ensure full reproducibility?

Partly

Is the study design appropriate and is the work technically sound?

Yes

Are the conclusions drawn adequately supported by the results?

Yes

Are sufficient details of methods and analysis provided to allow replication by others?

Yes

Reviewer Expertise:

Endometriosis and minimally invasive surgery

I confirm that I have read this submission and believe that I have an appropriate level of expertise to confirm that it is of an acceptable scientific standard, however I have significant reservations, as outlined above.

# Response

**Published:** 2024-08-09

**Authors:** Ann-Katrin Meyrose

**Affiliations:** Clinical Psychology and Psych, Helmut-Schmidt-University / University of the Armed Forces Hamburg, Germany

**Numbered Reviewer’s comments -->** ***Authors’ responses in italic (*** **Change in manuscript new word version in parentheses)**

 1. This is large study about patients expectation before a laparoscopic treatment of endometriosis. This point is very interesting. The methods used appear appropriate, but I am not a specialist of treatment expectations studies. 

 --> *Thank you very much for your valuable clinical perspective and suggestions on how to improve our manuscript.*

 2. I would suggest to include data about the anatomical severity of the disease. A global approach of endometriosis is in my mind always somewhat misleading. Treatment expectations from a laparoscopic procedure are likely very different from a patient who has a mechanical infertility related to adhesions to another one who has no disease at ultrasound and complains of severe pain. 

 --> *In this manuscript, we included people with suspected endometriosis who filled out our online questionnaire before laparoscopy. Unfortunately, we have no data about the anatomical severity of the disease, only limited and self-reported data for those participants.*

*A subsample of 395 people underwent laparoscopy and received an endometriosis diagnosis. We have more medical data (e.g., #ENZIAN, rASRM, deep infiltration) for that subsample, which was followed for one year after the laparoscopy. Two manuscripts are currently in progress, analyzing these medical and psychological factors in comparison.*

 3. Data about pre operative information and particularly about the risks of complications would be essential to understand these expectation. A patient "afraid of a bowel fistula" may have different expectation that patients with expected minimal or moderate disease based on preoperative imaging.

 When dealing with pain treatment these data would be very interesting. I would also recommend to collect data about the personal history of these patients, trauma and violence may influence the trust in treatment and the results of this very interesting study. I think that most of these data are available to the authors and could be added to the paper.

*--> We agree that the factors you mentioned likely shape patients’ expectations. Unfortunately, we have limited medical information for this large sample (please see comment above). However, we have added the following data we assessed to the manuscript: self-reported duration of symptoms, previous experience with laparoscopy, current endocrine therapy, and whether complaints are dependent on menstrual bleeding. Due to the limited space in the preoperative online questionnaire and the lack of previous evidence focusing on the expectations of patients with endometriosis, we decided to concentrate on assessing expectations through various measurements as well as disability, complaints, anxiety, depression, sociodemographics, and some medical characteristics.*

*To the best of our knowledge, our study is the first to investigate expectations in patients with endometriosis. To gain a deeper understanding of these expectations, we also conducted qualitative interviews before and after laparoscopy in an additional study module. The corresponding manuscript is currently under review, and if possible, we would be pleased to link it to this manuscript. As you suggested, patients reported that pre-operative information, fears, personal history, and prior experiences are important factors for them (among others). We added this aspect to the section “implications for practice and research”. (*Measurements – last paragraph p. 8; Results Table 2 and Descriptive characteristics page 9; Discussion section page 29)

*Finally, this manuscript has a methodological focus on expectations but we agree that the clinical perspective is also very interesting. We have added sentences to the discussion section of this manuscript to highlight the clinical perspective and the implications for future research.*

 (Discussion section, page 28/29)
